# A Novel Interaction between Pyk2 and MAP4K4 Is Integrated with Glioma Cell Migration

**DOI:** 10.1155/2013/956580

**Published:** 2013-09-15

**Authors:** Joseph C. Loftus, Zhongbo Yang, Jean Kloss, Harshil Dhruv, Nhan L. Tran, Daniel L. Riggs

**Affiliations:** ^1^Department of Biochemistry and Molecular Biology, Mayo Clinic Arizona, 13400 East Shea Boulevard, Scottsdale, AZ 85259, USA; ^2^Translational Genomics Research Institute, Phoenix, AZ 85004, USA

## Abstract

Glioma cell migration correlates with Pyk2 activity, but the intrinsic mechanism that regulates the activity of Pyk2 is not fully understood. Previous studies have supported a role for the N-terminal FERM domain in the regulation of Pyk2 activity as mutations in the FERM domain inhibit Pyk2 phosphorylation. To search for novel protein-protein interactions mediated by the Pyk2 FERM domain, we utilized a yeast two-hybrid genetic selection to identify the mammalian Ste20 homolog MAP4K4 as a binding partner for the Pyk2 FERM domain. MAP4K4 coimmunoprecipitated with Pyk2 and was a substrate for Pyk2 but did not coimmunoprecipitate with the closely related focal adhesion kinase FAK. Knockdown of MAP4K4 expression inhibited glioma cell migration and effectively blocked Pyk2 stimulation of glioma cell. Increased expression of MAP4K4 stimulated glioma cell migration; however, this stimulation was blocked by knockdown of Pyk2 expression. These data support that the interaction of MAP4K4 and Pyk2 is integrated with glioma cell migration and suggest that inhibition of this interaction may represent a potential therapeutic strategy to limit glioblastoma tumor dispersion.

## 1. Introduction

Glioblastoma multiforme (GBM) is the most common form of all primary adult brain tumors. Although significant technical advances in surgical and radiation treatments for brain tumors have emerged, their impact on clinical outcome for patients has been modest [1, 2]. Of the features that characterize GBM, arguably none is more clinically significant than the propensity of glioma cells to aggressively invade the surrounding normal brain tissue [3]. These invasive cells render complete resection impossible, confer strong resistance to chemo- and radiation therapy, and virtually assure the rise of secondary tumors that develop at the resection margins that drive further invasion [4]. Meaningful advances in clinical outcomes will require identification and targeting of key signaling effectors mediating glioma invasion.

 The nonreceptor tyrosine kinase proline-rich tyrosine kinase 2 (Pyk2) serves as a point of integration for signaling from cell surface receptors including integrin adhesion receptors, G-protein coupled receptors, and receptor tyrosine kinases [5–7]. As such, signaling from Pyk2 has been implicated in a variety of cellular processes including migration, cell survival, and proliferation. We have demonstrated in glioblastoma tumor samples that Pyk2 expression is upregulated in invasive glioma cells relative to cells in their cognate tumor cores [8] and that increased Pyk2 activity positively correlated with increased migration of glioma cells *in vitro* [9]. Furthermore, we established that increased expression of Pyk2 simulated glioma cell migration *in vitro* while specific knockdown of Pyk2 expression inhibited glioma cell migration *in vitro,* impaired invasion in organotypic brain slices, and increased survival and reduced invasion and distant tumor foci in an intracranial xenograft model [9, 10]. Specific inhibition of Pyk2 activity inhibited glioma cell migration *in vitro* [11] and prolonged survival in a xenograft model [11, 12]. Collectively, these data support Pyk2 as a potential target to inhibit glioblastoma invasion.

Pyk2 can be activated by integrin ligation [13, 14] and is activated in response to cellular stress and in response to a variety of agonists that raise intracellular calcium [7, 15, 16]. How agonist stimulation ultimately leads to Pyk2 activation remains unclear as the intrinsic mechanism of activation for this kinase remains to be defined. Pyk2 shares a conserved domain structure with the related focal adhesion kinase FAK including an N-terminal FERM domain, a central kinase domain, several proline rich domains, and a C-terminal focal adhesion targeting (FAT) domain. The FAT domain is critically involved in the activation of FAK by targeting FAK to the focal adhesion [17, 18]. Similarly, expression of FRNK, an alternatively spliced variant corresponding to the C-terminal portion of FAK, inhibits FAK activation by displacing FAK from the focal adhesion [19, 20]. Interestingly, although Pyk2 contains a highly conserved FAT domain and can be localized to the focal adhesion, it also exhibits a significant cytoplasmic distribution with perinuclear enrichment in a number of cell types suggesting that focal contact localization is not essential for Pyk2 activation. Indeed, substitutions within the Pyk2 FAT domain postulated to disrupt the four-helix bundle structure of the Pyk2 FAT domain [21] do not result in the loss of Pyk2 activity [22].

These data suggest that domains within Pyk2 other than the FAT domain may function to localize Pyk2 to specific cellular locations or in the regulation of Pyk2 activity. Among the candidates for functional regulatory domains in Pyk2 is the N-terminal FERM domain. FERM domains are compact protein modules comprised of three distinct subdomains found in a number of proteins. In the prototypical FERM domain proteins ezrin, radixin, and moesin, the FERM domains regulate their activity by mediating protein-protein interactions and membrane targeting. Previous studies support an important role for the N-terminal Pyk2 FERM domain in the regulation of Pyk2 function. We have demonstrated that intracellular expression of an autonomous Pyk2 FERM domain potently inhibited Pyk2 phosphorylation [9]. Subsequently, Kohno et al. [23] demonstrated that the FERM domain mediated the formation of Ca^2+^/calmodulin dependent Pyk2 homodimers that facilitated transphosphorylation. Structural analysis of several ligand-bound FERM domains has substantiated the importance of a surface formed by *β*5C-*α*1C of the F3 subdomain in ligand binding [24–30]. Select substitutions within this surface of the FERM domain of Pyk2 inhibit Pyk2 phosphorylation [11, 12]. Moreover, a monoclonal antibody targeting an epitope localized to the *β*5C-*α*1C surface of the F3 module of the Pyk2 FERM domain effectively inhibits Pyk2 phosphorylation when expressed as an intracellular scFv [12]. Together, these data support an important role for the FERM domain in the regulation of Pyk2 activity perhaps by mediating protein-protein interactions that are required for Pyk2 function. Similar studies into the regulation of activity of the closely related focal adhesion kinase FAK have provided compelling evidence for a functional role of the N-terminal FERM domain. Structural studies have demonstrated that the N-terminal FERM domain of FAK binds directly to the kinase domain thereby blocking access to the catalytic cleft [31, 32]. Lietha et al. [32] proposed that FAK activation results from displacement of the FERM domain, perhaps mediated by a protein-protein interaction with an activating protein. While the identity of the proposed activating protein remains to be determined, an important role for a conserved cluster of basic amino acids in the F2 subdomain has been described [33]. Importantly, we and others have been unable to demonstrate a similar interaction between the Pyk2 FERM domain and the Pyk2 kinase domain [33] suggesting that the mechanism of intrinsic activation is different for Pyk2 than for FAK. In the present study, we utilized yeast two-hybrid genetic selection to screen for novel protein-protein interactions mediated by the Pyk2 FERM domain. We identified the Ste20 homolog MAP4K4 as a Pyk2 binding partner and describe a role for integration of MAP4K4 with Pyk2 function in glioma cell migration.

## 2. Materials and Methods

### 2.1. Antibodies

The anti-FLAG M2 monoclonal antibody was from Sigma (St. Louis, MO). The rabbit anti-HA monoclonal antibody and the polyclonal anti-Pyk2 antibody were from Upstate Biotechnology (Lake Placid, NY). The anti-Pyk2 monoclonal antibody OT126 was from United States Biologicals (Swampscott, MA). The anti-MAP4K4 antibody was from Epitomics (Burlingame, CA). The anti-phosphotyrosine (pY) antibody pY20 was obtained from BD Transduction Laboratories (San Jose, CA). The IRDye conjugated secondary antibodies were from LI-COR Biosciences (Lincoln, NE). 

### 2.2. Yeast Two-Hybrid Selection

The strains, plasmids, and library used in the yeast two-hybrid (Y2H) interaction screen were obtained from Clontech (Mountain View, CA) and used as recommended by the manufacturer. The Y2H Pyk2 FERM bait plasmid was constructed by placing the Pyk2 FERM DNA (encoding Pyk2 E36 through A366) between the NcoI and BamHI sites of the bait vector pAS2-1. To select for Pyk2 FERM prey proteins the *Saccharomyces cerevisiae *strain, AH109 (*MATa, trp1-901, leu2-3, 112, ura3-52, his3-200, gal4D, gal80D*, *LYS:: GAL*1_*UAS*_ *- GAL*1_*TATA*_ *HIS3, GAL*2_*UAS*_ *- GAL*2_*TATA*_ *-ADE2, URA:: MEL*1_*UAS*_ *- MEL*1_*TATA*_ *-LacZ*) containing the Pyk2 FERM bait plasmid was transformed with a human fetal brain cDNA library (3.5 × 10^6^ independent clones) in the vector pACT2. The transformation reaction was plated onto SC plates minus His, Leu, and Trp (SC-His-Leu-Trp). After 4–6 days at room temperature, the colonies were replica plated onto the more stringent SC-Ade-His-Leu-Trp medium. Those colonies appearing on the stringent selection plate were purified and further analyzed. To confirm bait-prey interaction, *lacZ* reporter expression was assayed using the chemiluminescent *β*-galactosidase assay reagent Gal-Screen (Tropix, Bedford, MA) in 96-well microtiter plates following the manufacturer's instructions.

**Figure 1:**
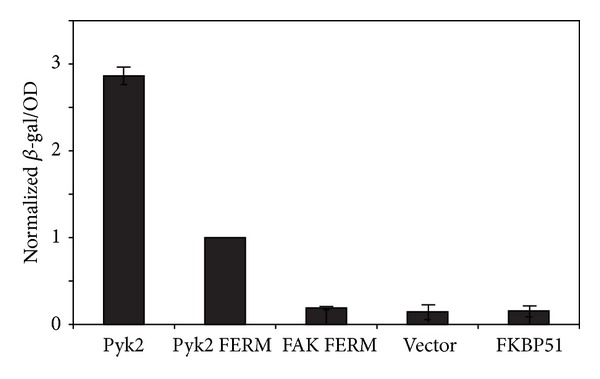
MAP4K4(143) interacts specifically with the Pyk2 FERM domain. Each strain contained MAP4K4(143) as prey and the indicated bait chimera. The bait-prey interaction was measured by dividing the *β*-galactosidase reporter activity (measured in relative light units, RLU) by the cell density (optical density, OD). This measure of specific *β*-galactosidase activity was normalized to the signal of the interaction of MAP4K4(143) with the Pyk2 FERM domain. Each bar is the average of two to four independent experiments. Error bars indicate the standard deviation.

The interaction of the clone MAP4K4(143) with various proteins was quantified by two-hybrid analysis in diploid strains (Figure 1) as follows. Strain Y189 (*MAT α, ura3-52, his3-200, ade2-101, trp1-901, leu2-3, 112, gal4Δ, met* ^−^ *, gal80Δ, URA3::GAL1uas-GAL1*_tata_ *-lacZ*) expressing clone MAP4K4(143) as prey was mated with strain AH109 harboring various bait chimeras by mixing the haploid strains on SC plates lacking leucine and tryptophan (SC-LW) and incubating the plates at 30°C for two days. The resulting diploid strains were cultured overnight in SC-LW broth, and then 100 *μ*L was assayed for *β*-galactosidase activity with Gal-Screen reagent. The relative light unit (RLU) signal produced by *β*-galactosidase was normalized for culture density (optical density, OD).

### 2.3. Cell Culture and Transfection

The human glioblastoma cell line SF767 and the 293T packaging cells were routinely passaged in DMEM (BioWhittaker, Walkersville, MD) containing 10% fetal bovine serum, 1% nonessential amino acids, 2 mM glutamine, 100 units/mL penicillin, and 10 *μ*g/mL streptomycin. Transfections of subconfluent cultures were performed with the Effectene reagent (Qiagen, Chatsworth, CA) as previously described [11]. 

### 2.4. Expression Constructs

The construction of the following expression plasmids has been previously described: the FLAG-epitope-tagged Pyk2, the FLAG-epitope-tagged kinase-deficient Pyk2 (K457A), and the HA-epitope-tagged Pyk2 FERM [9]; the HA-epitope-tagged FAK [34]; the HA-epitope-tagged Pyk2 FERM, and the HA-epitope-tagged FAK FERM domain encoding FAK residues R35-P362 [11, 12].

The MAP4K4 sequence contained in clone 143 isolated from the yeast two-hybrid screen (corresponding to MAP4K4 residues 929–1273 based on the numbering of isoform 2, the longest isoform; Accession NM_145686) was amplified by PCR and ligated in frame downstream of a 3X HA epitope in pcDNA or downstream of a 3X FLAG epitope in p3XFLAG-CMV (Sigma, St. Louis, MO). The coding sequence for full length MAP4K4 was isolated by RT-PCR of total RNA isolated from the SF767 glioma cell line using the Titan RT-PCR kit (Roche Applied Science, Indianapolis, IN) according to the manufacturer's instructions. The final clone contains alternatively spliced modules M1, M2, M3, and M8 [35] and was ligated in frame downstream of a 3X HA epitope in pcDNA. 

Two shRNAs directed against MAP4K4 were generated from siRNA sequences previously reported to knockdown MAP4K4 expression. The first shRNA, designated 1M4K4i, was based on the RNAi sequence described by Mack et al. [36] and had the following sequence: 5′-GATCCGTGGTTGGAAATGGCACCTttcaagagaAGGTGCCATTTCCAACCACTTTTTGGAAAA-3′. The second shRNA, designated 2M4K4i, was based on the RNAi sequence described by Collins et al. [37] and had the following sequence: 5′-GATCCGGGAAGGTCTATCCTCTTAttcaagagaTAAGAGGATAGACCTTCCCTTTTTGGAAAA-3′. Annealed shRNA oligonucleotide duplexes were ligated to BamHI/HindIII digested pSilencer 3.0-H1 (Ambion, Austin, TX) and the sequence verified by direct DNA sequencing. The H1 promoter and shRNA were excised from pSilencer by EcoRI/MluI digestion and ligated to the similarly digested lentiviral transfer vector pLVTHM (Addgene, Cambridge, MA). pLVTHM contains a separate transcriptional cassette in which the EF1-*α* promoter drives GFP expression. 

### 2.5. Lentiviral Transduction

VSV-G pseudotyped recombinant lentiviruses were produced by transient transfection of 293 packaging cells. Subconfluent cultures of 293 cells were transfected with 20 *μ*g of the appropriate LVTHM construct, 15 *μ*g of psPAX2 packaging plasmid, and 5 *μ*g of pMD2.G envelope vector by calcium phosphate precipitation. For lentiviral transduction, medium containing recombinant lentiviruses was harvested from the packaging cells after 48 hours, concentrated by PEG precipitation and centrifugation, and added to subconfluent cultures of target cells together with 8 *μ*g/mL polybrene for 4–6 hours at 37°C. Positively transduced cells were enriched by mass sorting the GFP positive cells on a Vantage flow cytometer (BD Biosciences, San Jose, CA). The generation of SF767 glioma cells stably transduced with a shRNA targeting Pyk2 has been previously described [10].

### 2.6. Radial Migration Assay

A monolayer radial migration assay was used as previously described [34, 38]. Briefly, 2500 control or transduced cells were plated/well of a Cell Sedimentation Manifold (Creative Scientific Methods, Phoenix, AZ) on laminin coated slides. Cells were incubated for 16 hours, the manifold removed, and a measurement of the diameter of the seeded cells was made with an inverted microscope and image analysis software (Scion Image, Frederick, MD). Linear migration from the initial seeded area was determined for 10 replicate samples 24 hours after removal of the manifold. Specific migration rates were calculated by normalizing the measurements to nonspecific migration on BSA. Migratory rates were calculated and group means determined.

### 2.7. Transwell Assay

Transwell assays were performed using a modified Boyden chamber (Neuroprobe, Cabin John, MD) as previously described [39]. Briefly, each well contains an 8 *μ*m pore size Nucleopore filter coated with 50 *μ*g/mL bovine collagen (PureCol, Advanced Biomatrix, Poway, CA). Control or transduced SF767 glioma cells were seeded at 4.8 × 10^4^ cells in 100 *μ*L of complete media (DMEM containing 10% fetal bovine serum) to the top well of the chamber, and complete media were added to the lower chamber. After incubation for 48 hours at 37°C, nonmigrated cells were scraped off the upper side of the filter, and filters were stained with 4′,6-diamidino-2-phenylindole (DAPI). Nuclei of migrated cells were counted in 5 high-power fields (HPF) with a 20x objective. Values were assessed in triplicate.

### 2.8. Cell Proliferation Assay

Control transduced SF767 cells or SF767 cells stably transduced with a shRNA targeting MAP4K4 (MAP4K4i) were seeded in triplicate in complete media. Cell proliferation was measured at 24-hour intervals for 5 days using the MTT assay according to the manufacturer's instructions (Sigma, St. Louis, MO).

### 2.9. Immunoblotting and Immunoprecipitation

Cells were washed in cold PBS, lysed by addition of 1 mL IPB buffer (137 mM NaCl, 20 mM Tris, pH 7.5, 1% NP-40, and 10% glycerol containing protease and phosphatase inhibitors), and incubated on ice for 30 minutes. Lysates were clarified by centrifugation at 16,000 ×g for 10 minutes at 4°C. Protein content of the lysate was determined using the BCA assay (Sigma). Immunoblotting and immunoprecipitation of cleared lysates was performed as described [11]. Detection was performed with HRP-conjugated secondary antibodies and enhanced chemiluminescence (Perkin Elmer Life Sciences, Boston, MA) or by infrared detection using IRDye conjugated secondary antibodies with the Odyssey Infrared Imaging System (LI-COR Biosciences, Lincoln, NE). Quantitation of band intensity was performed with the Odyssey application software v3.0.

### 2.10. Statistics

Migration data was analyzed using GraphPad Prism 5.0 (GraphPad Software, La Jolla, CA). Independent sample *t*-tests were used for analysis involving two samples and one-way analysis of variance for tests involving more than two samples. All tests are two tailed. Data are presented as the means ± SEM. Differences were considered significant at *P* < 0.05. 

## 3. Results

### 3.1. Identification of MAP4K4 as a Binding Partner for the Pyk2 FERM Domain

**Figure 2:**
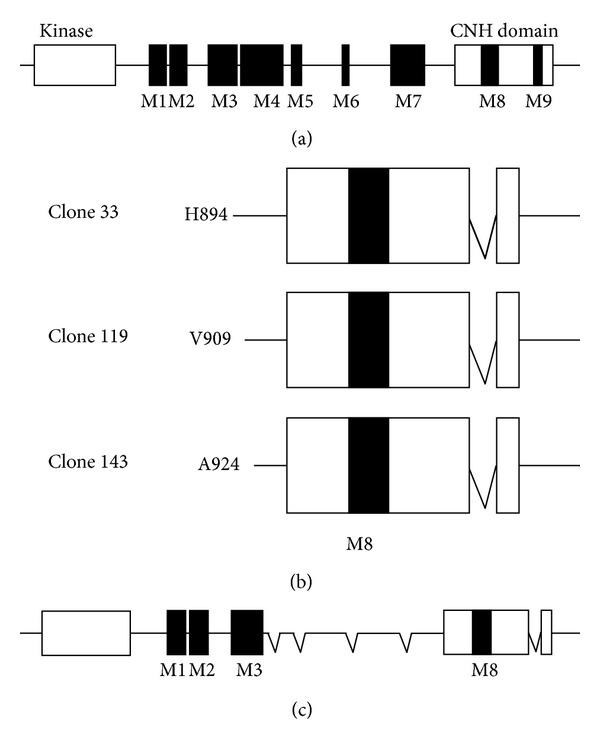
Schematic of MAP4K4 and interacting two-hybrid clones. (a) Structure of full length MAP4K4 indicating the N-terminal kinase domain, the C-terminal citron homology domain (CNH), and the location of alternatively spliced modules M1–M9. (b) Structure of the three overlapping clones isolated in the yeast two-hybrid genetic screen which interact with the Pyk2 FERM domain. (c) Structure of the final MAP4K4 clone assembled from SF767 glioma cell isoforms.

Our previous studies supported a role for the Pyk2 FERM domain in the regulation of Pyk2 activity and function. To identify proteins that interact with the Pyk2 FERM domain, we performed a yeast two-hybrid selection from a human fetal brain cDNA library using the Pyk2 FERM domain (encoding residues E36-A366) as the bait. Screening of 1.2 × 10^6^ transformants resulted in the isolation of clone 143 that interacted with the Pyk2 FERM domain. Sequence analysis indicated that clone 143 encoded the C-terminal one-third of MAP kinase kinase kinase kinase 4 (MAP4K4). This clone was designated MAP4K4(143). As we were interested in proteins that interacted specifically with the Pyk2 FERM domain, we examined the interaction of clone MAP4K4(143) with the closely related FAK FERM domain (FAK residues R35-P362) by two-hybrid analysis. Interaction of MAP4K4(143) with the FAK FERM domain was not significantly greater than the interaction of MAP4K4(143) with bait vector alone or with the unrelated bait FKBP51 (Figure 1). MAP4K4(143) also interacted strongly with full length Pyk2. The yeast two-hybrid selection also identified two additional independent, overlapping MAP4K4 clones, designated MAP4K4(33) and MAP4K4(119). The three overlapping clones, starting at codons H884, V909, and A924, all encompass the C-terminal citron homology domain (CNH) [40] of MAP4K4 and include the alternatively spliced module M8 but lack module M9 (Figure 2). 

**Figure 3:**
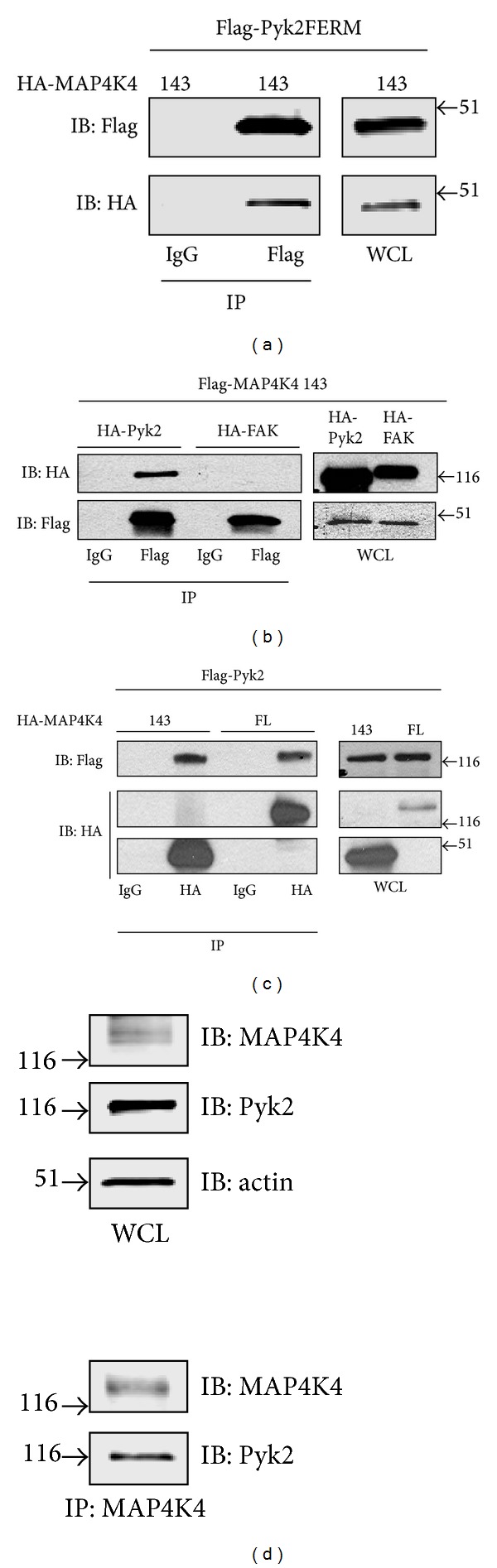
Pyk2 coimmunoprecipitates with MAP4K4. (a) Cells cotransfected with FLAG-epitope-tagged Pyk2 FERM and HA-epitope-tagged MAP4K4(143) were lysed and immunoprecipitated with anti-FLAG antibody or normal mouse IgG. Immunoprecipitates were immunoblotted (IB) with anti-FLAG or anti-HA. WCL: whole cell lysate. (b) Cells were cotransfected with FLAG-epitope-tagged MAP4K4(143) and either HA-tagged Pyk2 or HA-tagged FAK, lysed, and immunoprecipitated with mouse IgG or anti-FLAG antibody. Immunoprecipitates or WCL were immunoblotted as indicated. (c) Cells cotransfected with FLAG-tagged Pyk2 and either HA-tagged MAP4K4(143) or HA-tagged full length (FL) MAP4K4 were lysed and immunoprecipitated with rabbit IgG or anti-HA antibody. Immunoprecipitates or whole cell lysate (WCL) was immunoblotted with the indicated antibodies. (d) SF767 glioma cells were lysed and immunoprecipitated with anti-MAP4K4 antibody. Immunoprecipitates or whole cell lysate (WCL) was immunoblotted with the indicated antibodies.

To confirm that MAP4K4 (143) interacted with the Pyk2 FERM domain in the intracellular environment of cultured cells, cells were cotransfected with FLAG-tagged Pyk2 FERM domain and HA-tagged MAP4K4 (143). Immunoblotting of the anti-FLAG immunoprecipitate demonstrated that the HA-tagged MAP4K4 (143) coimmunoprecipitated with the Pyk2 FERM domain (Figure 3(a)). To probe the specificity of the interaction, we compared the interaction of MAP4K4 (143) with Pyk2 and FAK. Cells were cotransfected with FLAG-epitope-tagged MAP4K4 (143) and either full length HA-tagged Pyk2 or full length HA-tagged FAK. Immunoprecipitation of the cotransfected cell lysates with an anti-FLAG antibody indicated that Pyk2 coimmunoprecipitated with MAP4K4 (143) (Figure 3(b)). Consistent with the results of the two-hybrid interaction assay, an appreciable amount of FAK was not detected in the MAP4K4 (143) immunoprecipitate. 

These results indicated that the C-terminal portion of MAP4K4 containing the CNH domain, previously reported to function as a protein interaction domain [35, 41], interacted with Pyk2. To examine whether full length Pyk2 interacted with full length MAP4K4, we isolated MAP4K4 from the SF767 glioma cell line. RT-PCR of total RNA isolated from SF767 cells resulted in the identification of seven different splice isoforms in SF767 cells consistent with the previous observation that multiple forms of MAP4K4 can be observed in the same cell [35]. From these seven different isoforms, we assembled a full length MAP4K4 comparable to the MAP4K4 isoform previously cloned from a SNB19 glioma cell library [35]. The final HA-tagged full length MAP4K4 clone encoded 1,239 amino acids and contained the alternatively spliced modules M1, M2, M3, and M8 (Figure 2(c)). To examine the interaction of full length MAP4K4 with Pyk2, cells were cotransfected with FLAG-tagged Pyk2 and either HA-tagged MAP4K4(143) or HA-tagged full length MAP4K4. Cell lysates were immunoprecipitated with rabbit IgG as a control or with an anti-HA antibody (Figure 3(c)). Immunoblotting of the immunoprecipitates indicated that Pyk2 coimmunoprecipitated with both the C-terminal MAPK4(143) clone and with full length MAP4K4. Similarly, endogenous MAP4K4 coimmunoprecipitated with Pyk2 from SF767 glioma cells which express both MAP4K4 and Pyk2 (Figure 3(d)). Immunoblotting of SF767 cell lysates indicated the presence of several MAP4K4 species consistent with our RT-PCR results and the previous observation of multiple isoforms of MAP4K4 in the same cell [35].

### 3.2. Association of MAP4K4 with Pyk2 Alters MAP4K4 Phosphorylation

**Figure 4:**
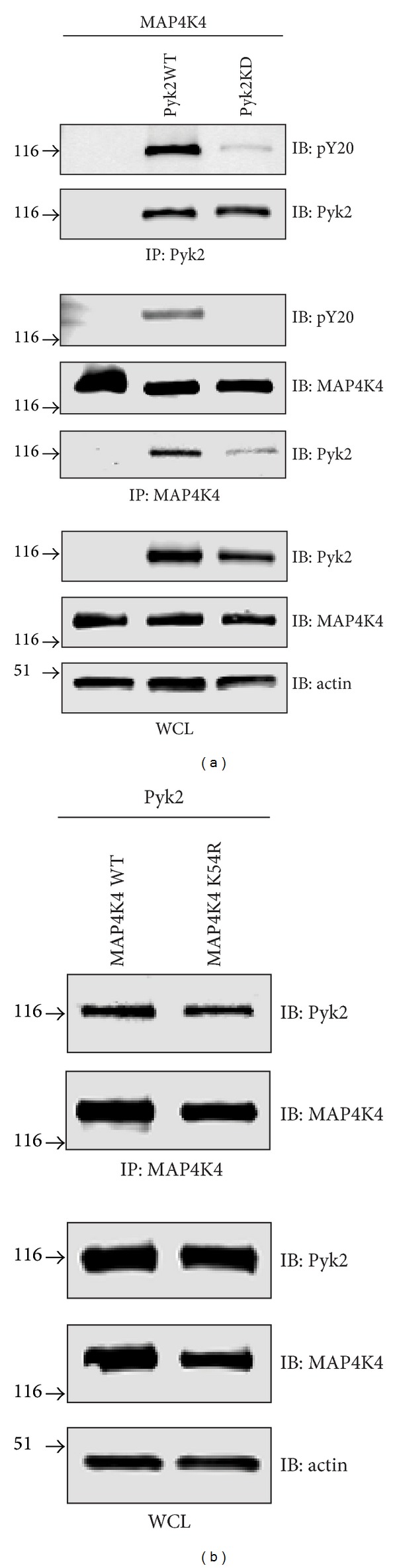
MAP4K4 is phosphorylated by Pyk2. (a) Cells were transfected with MAP4K4 or cotransfected with MAP4K4 and either wild type Pyk2 or a kinase-deficient Pyk2 variant (Pyk2KD). Cell lysates immunoprecipitated for Pyk2 were immunoblotted with anti-Pyk2 and the anti-phosphotyrosine antibody pY20. Cell lysates immunoprecipitated with anti-MAP4K4 antibody were immunoblotted for MAP4K4, anti pY20, and anti-Pyk2. Whole cell lysates (WCL) were immunoblotted with the indicated antibodies. (b) Cells were cotransfected with Pyk2 and wild type MAP4K4 or a kinase-deficient MAP4K4 variant (K54R). Anti-MAP4K4 immunoprecipitates or whole cell lysates were immunoblotted with the indicated antibodies.

As MAP4K4 coimmunoprecipitated with Pyk2, we examined whether this association altered MAP4K4 phosphorylation. Cells were cotransfected with HA-tagged MAP4K4 and either FLAG-tagged wild-type Pyk2 or a FLAG-tagged kinase-deficient form of Pyk2 (Pyk2KD). Cells were lysed, immunoprecipitated with an anti-Pyk2 antibody or an anti-MAP4K4 antibody, and the precipitates immunoblotted with the anti-phosphotyrosine antibody pY20 (Figure 4(a)). Immunoblotting of Pyk2 precipitated from cells cotransfected with wild-type Pyk2 and MAP4K4 demonstrated positive staining of Pyk2 with pY20. Immunoprecipitation of MAP4K4 from these cotransfected cells demonstrated that MAP4K4 co-immunoprecipitated with wild-type Pyk2 and was phosphorylated on tyrosine as indicated by positive pY20 staining. In contrast, immunoprecipitation of Pyk2 from cells cotransfected with a kinase-deficient Pyk2 and MAP4K4 demonstrated that only minimal pY20 staining was evident on the Pyk2KD variant verifying the loss of Pyk2 catalytic activity. No pY20 staining was observed on MAP4K4 immunoprecipitated from cells cotransfected with MAP4K4 and the kinase-deficient Pyk2. Notably, the loss of Pyk2 catalytic activity significantly reduced the amount of Pyk2 that coimmunoprecipitated with MAP4K4 suggesting that increased Pyk2 activity improved the association of MAP4K4 with Pyk2. Next, we examined the effect of MAP4K4 activity on the interaction with Pyk2 (Figure 4(b)). Cells cotransfected with wild-type Pyk2 and either wild-type MAP4K4 or a kinase-deficient MAP4K4 variant (K54A) were lysed and immunoprecipitated with an anti-MAP4K4 antibody. Analysis of the MAP4K4 immunoprecipitates indicated that equivalent amounts of Pyk2 co-precipitated with the wild-type and kinase-deficient MAP4K4 indicating that the catalytic activity of MAP4K4 did not appear to be required for its interaction with Pyk2.

### 3.3. Effect of Knockdown of MAP4K4 on Glioma Cell Migration

**Figure 5:**
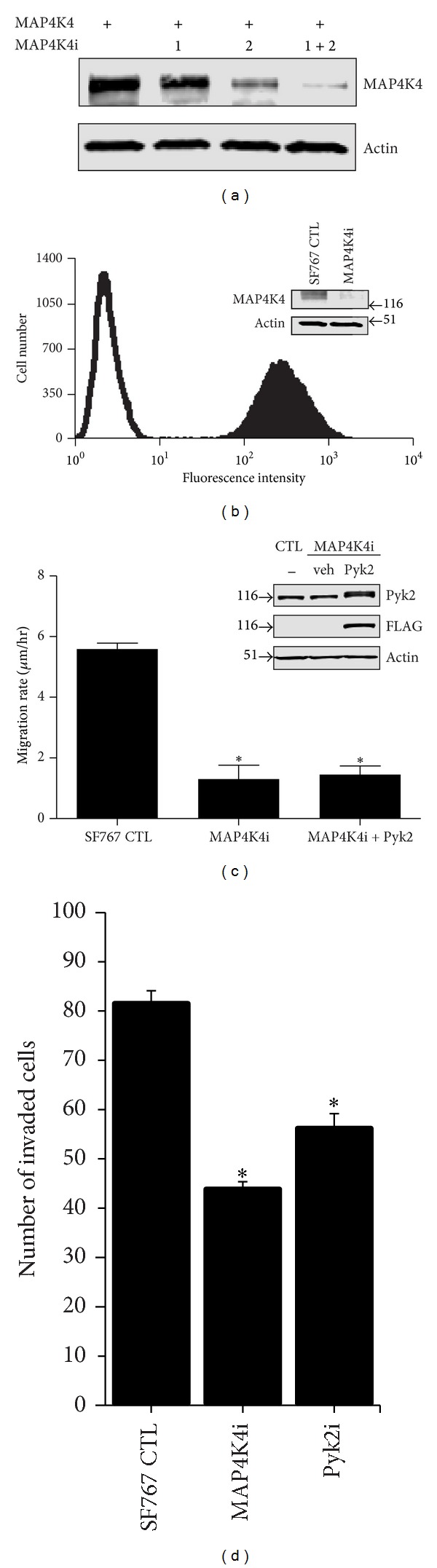
Knockdown of MAP4K4 expression inhibits glioma cell migration and invasion and blocks Pyk2 mediated stimulation of migration. (a) SF767 cells were transiently transfected with shRNA 1M4K4i and shRNA 2M4K4i or cotransfected with shRNAs 1M4K41i and 2M4K4i. Cell lysates were blotted with indicated antibodies. (b) SF767 cells were stably transduced with lentivirus expressing 2M4K4i shRNA. Stably transduced cells (solid histogram) were enriched by flow cytometry. Open histogram: control SF767 parental cells. Inset: whole cell lysates of control SF767 cells or SF767 cells stably transduced with MAP4K4 shRNA (MAP4K4i) were immunoblotted with the indicated antibodies. (c) The migration rate of control SF767 cells, SF767 cells stably transduced with a shRNA targeting MAP4K4 (MAP4K4i), and MAP4K4i cells transfected with Pyk2 was assayed with a radial migration assay on 10 *μ*g/mL laminin substrate. *P < 0.001 compared to control. (d) Transwell invasion assay of control SF767 cells, SF767 cells stably transduced with a shRNA targeting MAP4K4 (MAP4K4i), or SF767 cells stably transduced with a shRNA targeting Pyk2 (Pyki). *P < 0.001 compared to control.

To determine the effect of MAP4K4 expression on glioma cell migration, we examined the effect of knocking down expression of MAP4K4. Two different shRNAs, 1M4K4i and 2M4K4i, were tested for their capacity to knockdown MAP4K4 expression (Figure 5(a)). Transfection of SF767 cells with 1M4K4i produced a small reduction of MAP4K4 expression while transfection with 2M4K4i shRNA achieved a greater amount of knockdown. Cotransfection of cells with both shRNAs resulted in significant knockdown of MAP4K4 expression. The 2M4K4i oligonucleotide duplex was assembled into a lentiviral transfer vector for stable transduction of SF767 glioma cells. Transduced SF767 cells were enriched by mass sorting, and immunoblotting of the positively transduced cell population indicated significant reduction of endogenous MAP4K4 expression (Figure 5(b)). The effect of knockdown of MAP4K4 on the cell migration was examined by radial migration assay (Figure 5(c)). Knockdown of MAP4K4 expression significantly inhibited glioma cell migration. The observed reduction of migration in the MAP4K4 knockdown cells was not due to a reduction of proliferation as there was not a significant difference in the proliferation of MAP4K4 knockdown cells relative to SF767 control cells over the 24-hour time course of the migration assay as determined by MTT assay (data not shown). Knockdown of MAP4K4 expression also significantly inhibited glioma cell invasion in a transwell assay (Figure 5(d)).

We have previously shown that increased expression of Pyk2 stimulated glioma cell migration [9, 34]. To determine the effect of knockdown of MAP4K4 on Pyk2 stimulated glioma cell migration, SF767 cells with stable knockdown of MAP4K4 were transfected with FLAG-epitope-tagged Pyk2. Immunoblotting of cell lysates indicated that knockdown of MAP4K4 did not alter the expression of Pyk2. Notably, increased expression of Pyk2 in the MAP4K4 knockdown cells knockdown was unable to circumvent the inhibition of migration imposed by MAP4K4 knockdown. 

### 3.4. Silencing Pyk2 Expression Inhibits MAP4K4 Stimulated Glioma Cell Migration

**Figure 6:**
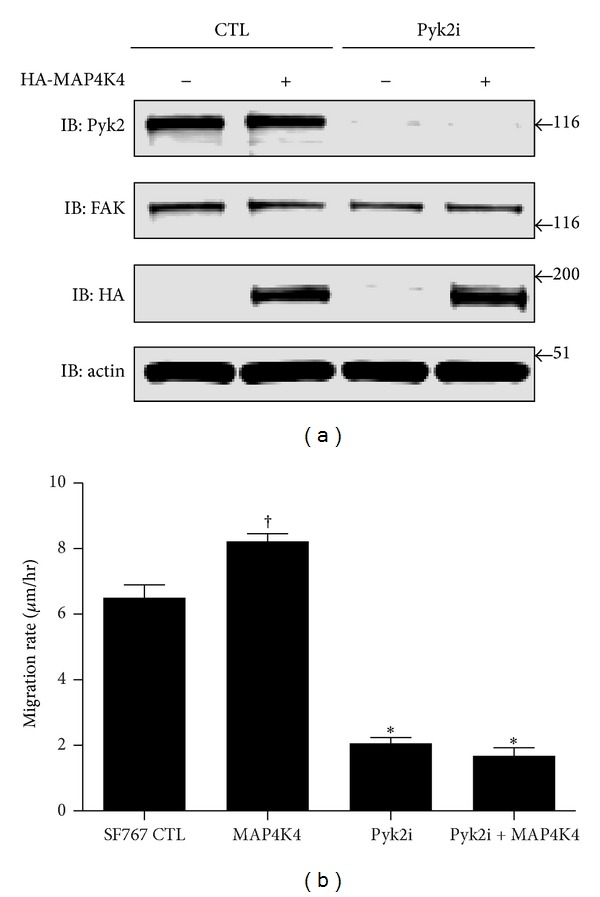
Increased expression of MAP4K4 stimulates migration but is blocked by knockdown of Pyk2 expression. (a) Wild-type SF767 glioma cells (CTL) or SF767 cells stably transduced with a shRNA targeting Pyk2 (Pyk2i) were transfected with vector or HA-epitope-tagged MAP4K4. Whole cell lysates were blotted with the indicated antibodies. Lysates were blotted with actin as a loading control. (b) SF767 control cells or SF767 cells with shRNA mediated knockdown of Pyk2 (Pyk2i) were transfected with MAP4K4, and the migration rate on 10 *μ*g/mL laminin was assessed over 24 hr using a radial migration assay. ^†^ *P* = 0.0016 relative to control. *P < 0.001 relative to control.

While increased expression of Pyk2 stimulated glioma cell migration in a dose-dependent manner, knockdown of Pyk2 expression by shRNA significantly inhibited glioma cell migration *in vitro*, invasion *ex vivo*, and increased survival of intracranial xenografts *in vivo* [9, 10]. To further examine the relationship between MAP4K4 and Pyk2 stimulated glioma cell migration, control SF767 glioma cells or SF767 glioma cells stably transduced with a shRNA targeting Pyk2 were transfected with MAP4K4, and the effect on cell migration was assayed with a radial migration assay (Figure 6). Increased expression of MAP4K4 in SF767 cells stimulated glioma cell migration relative to control SF767 cells. Consistent with previous studies [9], knockdown of Pyk2 expression significantly inhibited glioma cell migration. Notably, increased expression of MAP4K4 in the Pyk2 knockdown cells did not rescue glioma cell migration. 

## 4. Discussion

In previous studies, we have described a role for the Pyk2 FERM in the regulation of Pyk2 function. Expression of an autonomous Pyk2 FERM domain inhibited Pyk2 phosphorylation and Pyk2 stimulated glioma cell migration *in vitro* and increased survival in an intracranial xenograft model [10]. Substitution of residues that map to a shallow groove on the surface of the F3 subdomain of the Pyk2 FERM or targeting this surface with a monoclonal antibody similarly inhibited Pyk2 phosphorylation and function [12]. These data suggest that Pyk2 FERM domain mediated interactions are important for Pyk2 function in glioma cell migration. In the present study, we sought to identify novel protein-protein interactions mediated by the Pyk2 FERM domain. The major findings of this report are as follows: (1) the C-terminal CNH domain of the Ste20 homolog MAP4K4 was identified as a binding partner for the Pyk2 FERM domain, (2) the MAP4K4 CNH domain and full length MAP4K4 coimmunoprecipitated with Pyk2 from cell lysates, (3) knockdown of MAP4K4 expression inhibited glioma cell migration and blocked Pyk2 stimulation of migration, (4) increased expression of MAP4K4 stimulated glioma cell migration, (5) MAP4K4 stimulation of glioma cell migration was blocked by knockdown of Pyk2 expression. Together these data suggest a role for the integration of MAP4K4 and Pyk2 in glioma cell migration.

Increased intracellular expression of Pyk2 is accompanied by increased Pyk2 phosphorylation. Although the exact mechanism for this increased phosphorylation remains to be defined, we have demonstrated that increased expression of Pyk2 results in the formation of Pyk2 oligomers that facilitate transphosphorylation of Pyk2 and the recruitment of Src [22]. It has been reported that increased intracellular Ca^2+^ induces the formation of Ca^2+^/calmodulin dependent Pyk2 homodimers resulting in increased Pyk2 phosphorylation [23]. Mutations within a putative Ca^2+^/calmodulin binding site in F2 subdomain of the Pyk2 FERM significantly reduced the formation of Pyk2 homodimers and Pyk2 phosphorylation. Consistent with our previous results [11], it was also demonstrated that expression of an autonomous FERM domain inhibited Pyk2 phosphorylation through the formation of a heterodimer between the FERM domain and full length Pyk2 blocking the formation of Pyk2 homodimers. These data demonstrate the mechanistic importance of Ca^2+^ binding to the FERM domain to the activation of Pyk2. We have demonstrated that mutations within the F3 subdomain of the Pyk2 FERM domain result in the loss of Pyk2 phosphorylation [12]. Notably, the effect of these F3 mutations is independent of the capacity of Pyk2 to form oligomers suggesting that in addition to its requirement for binding Ca^2+^/calmodulin, the FERM domain might mediate protein-protein interactions that are required for protein re-arrangement and efficient phosphorylation [22]. The FERM domain is well appreciated as a protein-protein interaction domain [42], and the Pyk2 FERM domain has been reported to interact with several proteins including Nir1, FIP200, and the tumor suppressor p53 [43–45]. The relationship of the interaction of Pyk2 with these proteins to the regulation of Pyk2 kinase activity or in its larger role as a protein scaffold remains an area of active investigation.

That the Pyk2 FERM domain is involved in the regulation of Pyk2 activity and could act as a scaffold for functionally important interactions led us to look for specific protein interactions mediated by the FERM domain. Utilizing a yeast two-hybrid selection assay and the Pyk2 FERM domain as the bait, we identified three overlapping clones that corresponded to the C-terminal CNH domain of MAP4K4. Interestingly, these clones did not interact significantly with the FERM domain from the related FAK kinase in the yeast interaction assay nor did they co-immunoprecipitate with FAK from cell lysates. In contrast, the CNH domain containing MAP4K4(143) clone coimmunoprecipitated with the Pyk2 FERM domain as well as full length Pyk2 from cell lysates. The CNH domain was first described in citron Rho-interacting kinase [40] where it mediates an interaction with Rho GTPases [46]. Notably, this domain has been reported to mediate the interaction of murine MAP4K4 with MEKK1 and the cytoplasmic domain of integrin *β*_1_ linking MAP4K4 to cytoskeletal organization and cell migration [41, 47].

MAP4K4 is expressed at low levels in normal tissues but highly expressed in brain [41, 48]. MAP4K4 is overexpressed in many tumor cell lines, has been shown to modulate cellular transformation, invasion, and adhesion, and is highly expressed in GBM tumor samples [35]. Several compelling lines of evidence support MAP4K4 as a potential driver of cell migration. MAP4K4 was identified as a promigratory kinase in a genome wide siRNA screen for modulators of tumor cell motility [37] and is part of a five-gene signature that is a positive predictor of metastasis and negative survival in colorectal cancer [49]. Knockout studies in *Drosophila*, *C. elegans*, and mice demonstrated that MAP4K4 is essential for proper cell migration in embryonic development [47, 50, 51]. Interestingly, the phenotype of the MAP4K4^−/−^ mouse shares significant similarities to the fibronectin^−/−^ and integrin *α*_5_^−/−^ knockout mice suggesting a potential role for MAP4K4 in adhesion receptor signaling pathways [51] consistent with its interaction with *β*_1_ integrin. Notably, MAP4K4 expression is upregulated by EGFRvIII expression in glioma cells [52], and it is known to interact with the FERM domain of classical ERM proteins and phosphorylates them to promote F-actin anchoring and stabilization of lamellipodia in response to EGF and PDGF [53]. MAP4K4 also binds to the SH3 domain of the adapter protein Nck [41] promoting actin assembly and membrane protrusion [54, 55]. We confirmed the interaction of MAP4K4 with Pyk2 by co-immunoprecipitation suggesting that MAP4K4 may play a role in Pyk2 stimulated migration of glioma cells. Association of MAP4K4 with Pyk2 altered the phosphorylation of MAP4K4 as MAP4K4 that coimmunoprecipitated with wild-type Pyk2 was tyrosine phosphorylated. In contrast, phosphorylation of MAP4K4 was not observed when MAP4K4 was immunoprecipitated from cells cotransfected with a kinase deficient Pyk2. The functional significance of this phosphorylation is unknown and an area of current investigation. 

Knockdown of MAP4K4 significantly inhibited glioma cell migration and effectively blocked Pyk2 stimulated glioma migration suggesting that MAP4K4 may integrate with Pyk2 signaling. Increased expression of MAP4K4 stimulated glioma cell migration consistent with the previous observation that increased expression of MAP4K4 in rat intestinal epithelial cells reduced cell spreading and adhesion and increased cell invasion through Matrigel [35]. Interestingly, increased expression of MAP4K4 was unable to rescue the inhibition of glioma cell migration following Pyk2 knockdown suggesting that an interaction between Pyk2 and MAP4K4 is integrated with the stimulation of glioma cell migration. Alternatively, the interaction of Pyk2 and MAP4K4 may take place within the context of a larger protein scaffold of signaling effectors involved in cell migration. In this regard, it has been reported that the small GTP-binding protein Rap2 interacts with MAP4K4 and enhanced MAP4K4 mediated JNK activation [56]. Increased JNK activation has been linked to the pathobiology of glioblastoma [57–59]. It has also been reported that Rap activation downstream of integrin engagement induced Pyk2 phosphorylation [60]. Thus, Pyk2 could function as a component of a protein scaffold to colocalize Rap and MAP4K4 and promote translation of receptor mediated adhesive interaction into changes in actin polymerization and migration. Future studies will seek to identify the signaling pathways associated with this interaction and to substantiate the requirement for this interaction in glioma cell invasion in orthotopic mouse models.

## 5. Conclusion

In summary, we report a novel interaction between MAP4K4 and Pyk2. The interaction of MAP4K4 with Pyk2 appears to be part of a signaling pathway associated with glioma cell migration. Increased expression of MAP4K4 stimulated glioma cell migration that was blocked by knockdown of Pyk2 expression. Conversely, knockdown of MAP4K4 expression significantly inhibited glioma cell migration that could not be rescued by increased Pyk2 expression. Therefore, the current results suggest that inhibition of the interaction of MAP4K4 and Pyk2 may represent a potential therapeutic strategy to limit glioblastoma tumor dispersion.
